# Coronal Discoloration Induced by Calcium-Enriched Mixture, Mineral Trioxide Aggregate and Calcium Hydroxide: A Spectrophotometric Analysis

**DOI:** 10.7508/iej.2016.01.005

**Published:** 2015-12-24

**Authors:** Behnaz Esmaeili, Homayoun Alaghehmand, Tavoos Kordafshari, Ghazaleh Daryakenari, Maryam Ehsani, Ali Bijani

**Affiliations:** a * Dental Materials Research Center, Department of Operative Dentistry, Dental School, Babol University of Medical Sciences, Babol, Iran;*; b *Department of Operative dentistry, Dental School, Golestan University of Medical Sciences, Golestan, Iran; *; c *Department of Endodontics, Dental School, Babol University of Medical Sciences, Babol, Iran;*; d *Health Research Center, Babol University of Medical Sciences, Babol, Iran*

**Keywords:** Calcium-Enriched Mixture Cement, Mineral Trioxide Aggregate, Spectrophotometry, Tooth Discoloration

## Abstract

**Introduction:**

The aim of this study was to compare the discoloration potential of calcium-enriched mixture (CEM) cement, white mineral trioxide aggregate (WMTA) and calcium hydroxide (CH), after placement in pulp chamber.

**Methods and Materials:**

Access cavities were prepared in 40 intact maxillary central incisors. Then, a 2×2 mm box was prepared on the middle third of the inner surface on the buccal wall of the access cavity. The specimens were randomly assigned into four groups; the boxes in the control group were left empty, in groups 1 to 3, the boxes were filled with CH, WMTA and CEM cement, respectively. The access cavities and the apical openings were sealed using resin modified glass ionomer (RMGI). The color measurement was performed with a spectrophotometer at the following intervals: before (T0), immediately after placement of the filling material (T1), one week (T2), 1 month (T3), 3 months (T4) and 5 months (T5) after filling of the box and finally immediately after removing the material from the boxes (T6). Color change (*ΔE*) values were calculated using the sample Kolmogorov-Smirnov test to determine the normal distribution of data, followed by ANOVA, repeated measured ANOVA and post-hoc Tukey’s tests.

**Results:**

All materials led to clinically perceptible crown discoloration after 1 week. The highest *ΔE* value belonged to WMTA group. Discoloration induced by CEM cement was not significantly different from CH or the control group (*P*>0.05).

**Conclusion:**

CEM cement may be the material of choice in the esthetic region, specifically pertaining to its lower color changing potential compared to WMTA.

## Introduction

Common endodontic cements induce discoloration of the treated teeth, which will impair the esthetical outcome of the treatment [1, 2]. Although the mechanism of this noticeable color change is not clear yet, it is suspected to be the result of material ingression into the dentinal tubules. It is seen however, that visible crown discoloration, may not always be a result of tubular penetration; the remnants of therapeutic materials in the pulp chamber may also reflect a dark shade through translucent, hard tissues of the tooth such as the enamel, which tends to get darker in time [1, 3, 4].

Calcium-enriched mixture (CEM) cement, mineral trioxide aggregate (MTA) and to a lesser degree, calcium hydroxide (CH), are some examples of biomaterials used in treatment of the traumatically exposed pulps of immature teeth. The aim of such treatment is to preserve pulpal vitality and consequently allow physiological advancement and lateral dentin development of the root and induce apex closure. Thus, cervical root fractures, due to thin dentinal walls, are prevented [5, 6].

CH offers antimicrobial properties and improbability of dental discoloration, and is thus used as an intra-canal medication [7-9]. Modification of its formulation, to increase antimicrobial properties, radiopacity, *etc.*, has however demonstrated different staining potentials [1]. Crown discoloration is reported in some studies after using commercial CH preparations and CH containing sealers [3, 4, 10-12].

MTA has proven to be more successful in comparison with CH by overcoming its disadvantages [5, 13-15]. It is a bioactive silicate, containing hydrophilic particles, such as tricalcium silicate, tricalcium aluminate, dicalcium silicate, tricalcium oxide, bismuth and iron [16]. Original MTA was first introduced in grey color, which caused severe discoloration after its application and concerns were raised about the aesthetic competency of this material. The tooth colored formula was then introduced to compensate for this short coming [5, 17]. The crystalline structure as well as the chemical properties and mechanism of action is somewhat unchanged, although higher concentrations of aluminum oxide, magnesium oxide and iron oxide in grey MTA, result in a considerable staining potential [17, 18].

Despite all efforts, there are still reports on tooth discoloration after application of both kinds of MTA [18-20]. Moreover, MTA is an expensive material with a delayed setting time and poor handling characteristics. To overcome the mentioned disadvantages, CEM cement is introduced. Less than 1 h setting time, bactericidal effect against endodontic pathogens and alkaline pH (>10.5), makes this material a potent pulpal medicament. CEM cement powder is majorly composed of CaO, SO_3_, P_2_O_5_ and SiO_2_, with a small amount of Al_2_O_3_, Na_2_O_3_, MgO and Cl [15]. Evidence shows that CEM cement, despite antibacterial properties similar to CH, is more biocompatible and has a higher potential in hard tissue induction after vital pulp therapy [21, 22].

The aim of this *in vitro* study was to evaluate the probable chromatic alterations, after using MTA, CEM cement and CH in pulp chamber of extracted human central maxillary incisors.

## Materials and Methods

Forty extracted intact human central maxillary incisors, were collected. The teeth were free of caries, restorations or coronal discoloration. All the specimens were stored in 0.05% thymol solution for disinfection and they were cleaned with an ultrasonic device and then rubber cap and pumice to remove debris and extrinsic stains, from the crown surface. After that, the teeth were kept in saline solution.

Specimens were sectioned in the coronal third of the root complex, 1 mm below the buccal CEJ level. An access cavity was prepared, using a #4 round bur (Teezkavan Co., Tehran, Iran). Barbed brouches (Dentsply, Tulsa Dental, Tulsa, OK, USA) were used to remove the pulp tissue remnants from the access cavity.

To place the intended materials in the pulp chamber, a 2×2 mm box was prepared in the middle third of the lingual surface of the buccal wall, using a small fissure bur (Teezkavan Co, Tehran, Iran). This would leave a 2 mm thickness of tooth structure on the labial wall, which was measured for each specimen, using a caliber.

Teeth were then blindly divided into four groups (*n*=10): The control group; which had no material placed in the box; and in other groups either CH (Cinabartar, Tehran, Iran), tooth-colored MTA (Angelus, Londrina, Paraná, Brazil) and CEM cement (BioniqueDent, Tehran, Iran) were placed in prepared boxes.

All materials were prepared according to the manufacturer’s instructions. After setting of the materials, the coronal and apical openings were sealed, using resin modified glass ionomer (Fuji II LC, GC, Japan). The specimens were kept individually in artificial saliva containing tubes, in room temperature. The saliva solution was replenished every two weeks.

Spectrophotometer (VITA Easyshade; VITA Zahnfabrik, Bäd Sackingen, Germany) was used to measure the color changes, in a standardized condition. The lingual surface of each crown was fixed with a silicone mass. Another silicone impression was used to register the position of the spectrophotometer head on the buccal surface of the tooth. The measurements were carried out in the same room every time.

To repeat the exact measuring point, a silicone impression was made from the head of the spectrophotometer, which was used to replicate the acrylic model of it. This model was then fixed on the middle third of the teeth, followed by a silicone index of its location, to mark the point where the measuring tip must be placed each time.

The instrument was calibrated before measurement in each group. Color measurement was done at seven sessions obtained at the following intervals: prior to filling the box (T0=baseline), immediately after placement of the filling (T1), after one week (T2), one month (T3), 3 months (T4), 5 months (T5), and finally immediately after removal of the material from the access cavity (T6). Gentle air-drying was applied for 1 sec, each time before the measurements, to prevent optical changes caused by dehydration.

The measurements were repeated three times for each specimen and the mean values were calculated. The calculation of the color variation (*ΔE*) between the two color measurements was done as follows: *ΔE*=[(*ΔL*)2+(*Δa*)2+(*Δb*)2] [18]. *L* is a reference to the lightness coordinate, ranging from zero (black) to 100 (white), *a* and *b* are chromatic coordinates in the red-green axis and the yellow-blue axis, respectively [23].

Color matching limit, adopted in this study was 3.3 *ΔE* units (threshold of perceptibility). Differences beyond this were considered clinically perceptible [17]. The mean of the *ΔE* values were calculated in the given time intervals.

The obtained data was statistically analyzed using the one sample Kolmogorov-Smirnov test to determine the normal distribution, followed by ANOVA, repeated measures ANOVA and post-hoc Tuckey’s tests. The level of significance was set at 0.05.

**Figure 1:**
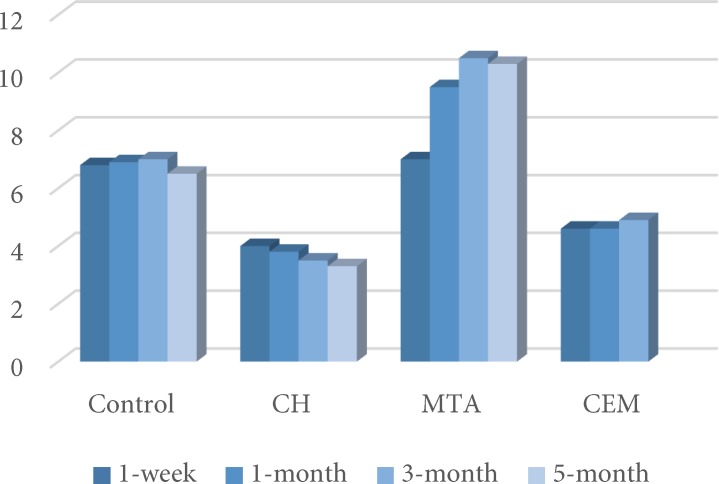
Comparing the mean *∆E* values between the four groups in T2. The red line indicates the clinical unacceptable color change perceptible by the viewer

## Results

Discoloration was evident in all teeth after the first week (T2) (Figure 1). MTA induced significantly more severe discoloration compared to other groups (*P*<0.05). For this group, the mean *ΔE* values followed an increasing pattern until the third month, and stayed stable from that time on. Total color change induced by MTA exceeded the perceptibly threshold (3.3), at all intervals. This group was the only group with significant discoloration (*P*=0.001).

In the other groups, the color change was higher than that of the perceptibility threshold in the first week, but did not have any significant elevations until the end of the experiment.

Color changes in the CEM cement group, had statistically no significant difference with the control and CH groups, but showed lower discoloration compared to MTA (*P*<0.05). CH and CEM cement groups, presented similar results in all measurement intervals (*P*>0.05).

**Figure 2:**
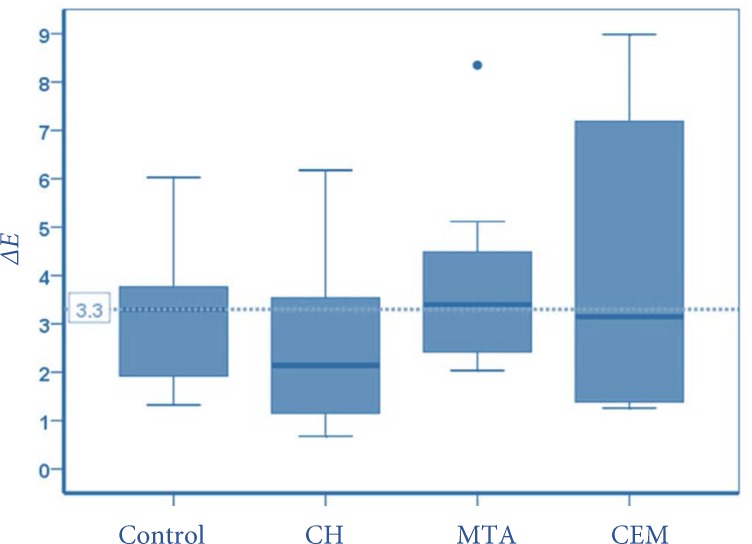
Distribution of *∆E* values, in T6 compared to T0. The green line indicates the clinical unacceptable color change perceptible by the viewer

After the removal of the materials, there was no significant difference in the *ΔE* values (T0-T6) in the study groups. According to Figure 2, more than 50% of the MTA specimens, had a clinically perceptible *ΔE*. It was however lower than 50% of the specimens for the other groups.* L*, *a* and *b* parameters were elevated after removing MTA, while in CEM cement and CH groups, *L* and to a lower portion *a* and *b* parameters were decreased (Tables 1 to 3).

## Discussion

The results indicate that all the materials used in this study, caused significant color changes, only one week after placement. The discoloration induced by CEM cement was significantly lower than MTA (*P*<0.05), in all intervals but was similar to that of the control and CH groups. The VITA Easy shade device was chosen in this study, due to its superiorities such as high data stability and replication excellency [1]. 

MTA samples presented rather severe discoloration compared to the other groups. MTA powder contains fine hydrophilic particles, which set in the presence of moisture. As reported by several investigations, the main elements of MTA are calcium, silica and bismuth oxide [24]. It has many beneficial characteristics, such as hard tissue conduction and induction and biocompatibility [18]. It is however, known to have some drawbacks, including handling difficulties, prolonged setting time, high cost, and unavailable known solvent, and above all discoloring of the dental structure [25]. Application of MTA for procedures, such as pulp therapy, apexification, treatment of internal root resorption and revascularization, often develops a macroscopic dark stain in the aesthetic zone [5, 26, 27]. The first manufactured MTA was grey. To compensate for the discoloring potential of original MTA, tooth-colored formula of MTA was introduced, with the same purpose of application [27]. Lower concentrations of carborundum (Al_2_O_3_), periclase (MgO) and specially FeO, are the major differences of WMTA with the former grey form. Higher amounts of FeO seem to be the primary culprit for the increased staining potential of grey MTA [28].

Despite the harmonious color of WMTA with dental tissues, tooth discoloration was observed in the findings of this study, which was in accordance with the results of some other studies [1, 17, 29]. These findings limit applications of MTA in endodontic treatments such as pulp capping and pulpotomy in the esthetic zones [18]. In the present study, the color change was evident in the first week and increased through the third month; however it remained stable from then until the 5^th^ month (Figure 1).

Variable amounts and durations of color change was reported with the same formulation of MTA, which would be the result of different thickness of the remaining tooth structure, colorimetric method of color measurement and material application methods [4, 17, 30]. Bortoluzzi *et al. *[31] however, reported no tooth discoloration, 6 months after replacing grey MTA with WMTA. The mentioned metal oxides, are present in the tooth-colored formula although to a very low degree, and can induce tooth discoloration [17]. Elements such as Fe, Mn and Cu, with d-electrons, are well known to have strong colors in oxide form. The d-electrons are readily excited by a visible spectrum light. Other oxides without such electrons (Ca, Si, Al, Mg and S) tend to be colorless or white, but the heavy ones such as bismuth has a yellow oxide [29]. Some authors state that the yellow oxide of bismuth used in both formulations of MTA for radiopacity, is the significant factor for tooth discoloration [32].

Another explanation about discoloration potential of WMTA, may be its increased solubility compared to grey MTA [33]. In clinic, WMTA is usually used directly in contact with vital, vascularized tissues of the pulp. Since the microstructure of MTA shows pH-dependent porosities, these may absorb blood components and result in discoloration [34]. Having in mind that there was no blood present in the tested specimens, macroscopic color examination of WMTA mass, after 5 months, revealed a dark blue to black color alteration. Simultaneously considerable improvement of the tooth color was seen immediately after removal of MTA. The alterations in* L*, *a* and *b* parameters revealed a significant increase in T6. This indicates an increase in the lightness and reduction in green and blueness. It can be concluded that clinically visible color change, occurring over time, is a reflection of the color change of the material itself, through dentin [5]. The results indicate that after one week, CH induced a color change that was above the threshold for perception. This discoloration was not significantly different from CEM cement and was very lower than MTA. 

CH used to be the gold standard for vital pulp therapy in 1930s [5]. Its low-grade irritation of the traumatized pulp tissue and the antibacterial effect makes CH the ideal material for pulp capping. There are detriments to this material as well, such as slow stimulation of dentinal bridge formation, gradual degradation, tunnel defect in the dentinal bridge and poor sealing properties [6, 13]. Not much data is available on the staining abilities of CH. The results of those who have reported dental color change after CH application, were in accordance to our findings [4, 10]. An investigation by Tinaz *et al. *[12], about the staining potential of CH treatment after removing AH-26 sealer from the root canal, concluded that using this material caused progressive discoloration of the teeth. Different CH intra-canal dressings had different discoloration capacities. The constituents that are added to CH may cause different staining potentials in pastes [1]. 

Allgayer and Lenherr [1], investigated the tooth color change after applying either injectable or pure CH. They reported color change in the injectable form which was due to bismuth carbonate, while the pure form didn’t present such a change. Their color measurements were however different from the present study. The data of this study indicate that after 5 months, CH did not induce visible color change and it was still white and opaque. Also, after removing the material (T6), chromatic parameters (*L* and to a lower degree, *a* and *b*) were decreased in comparison to T5, meaning that the lightness, yellowness and redness of the teeth, reduced which resulted in a tendency towards green and blue color change. This color change might be the result of the white shade of CH but no similar studies confirmed this.

**Table 1:** Mean (SD) values of *L*, *a* and *b* chromatic parameters at start point (T0) after placement of the material (T1), after one week (T2), one month (T3), 3 months (T4), 5 months (T5), and finally immediately after removal of the material from the access cavity (T6)

***L*** ** parameter**							
**Groups (N)**	**T0**	**T1**	**T2**	**T3**	**T4**	**T5**	**T6**
**Control (10)**	81.82 (5.29)	85.74 (5.03)	81.93 (4.31)	82.07 (4.37)	81.94 (3.94)	82.81 (4.58)	82.75 (4.72)
**CH (10)**	83.14 (7.07)	87.24 (6.54)	85.48 (5.4)	85.25 (5.25)	85.88 (5.18)	86.42 (5.54)	83.18 (6.57)
**MTA (10)**	82.09 (5.04)	86.53 (5.07)	83.63 (5.2)	81.92 (5.8)	80.25 (5.92)	80.91 (6.11)	82.23 (5.30)
**CEM Cement (10)**	80.04 (6.13)	83.81 (5.99)	82.57 (5.35)	83.47 (5.29)	83.14 (5.52)	83.70 (5.23)	81.85 (5.85)
***a*** ** parameter**							
**Groups (N)**	**T0**	**T1**	**T2**	**T3**	**T4**	**T5**	**T6**
**Control (10)**	-0.46 (1.28)	0.14 (1.41)	-0.90 (0.99)	-0.97 (0.97)	-0.88 (1.00)	-1.04 (0.98)	-1.18 (1.03)
**CH (10)**	-0.70 (0.77)	-0.15 (0.93)	-0.73 (0.9)	-0.73 (0.90)	-0.59 (0.86)	-0.85 (0.94)	-1.30 (0.77)
**MTA (10)**	-0.37 (1.22)	-0.18 (1.49)	-0.91 (1.09)	-1.1 (0.8)	-1.23 (0.7)	-1.36 (0.71)	-1.05 (0.91)
**CEM Cement (10)**	-0.15 (0.85)	0.39 (0.86)	-0.41 (0.97)	-0.37 (0.95)	-0.42 (0.93)	-0.64 (0.94)	-0.71 (0.93)
***b*** ** parameter**							
**Groups (N)**	**T0**	**T1**	**T2**	**T3**	**T4**	**T5**	**T6**
**Control (10)**	26.80 (5.5)	29.74 (5.16)	24.58 (4.69)	24.45 (5.06)	24.3 (4.95)	24.12 (4.98)	24.32 (5.24)
**CH (10)**	24.3 (3.65)	26.74 (4.42)	23.9 (4.02)	24.24 (3.86)	24.61 (3.98)	24.72 (3.98)	23.03 (3.68)
**MTA (10)**	23.55 (5.56)	27.22 (5.86)	21.49 (5.32)	19.38 (4.9)	19.19 (4.35)	18.97 (4.77)	20.72 (4.96)
**CEM Cement (10)**	25.38 (3.25)	28.5 (3.48)	24.85 (3.07)	24.35 (3.2)	24.33 (3.3)	23.98 (3.18)	23.65 (3.36)

CEM cement has clinical applications similar to MTA, but its composition is different. Apart from other good physical properties (*e.g.* less than 1 h setting time and thinner film thickness compared to MTA), it is capable of hydroxyl apatite formation in normal saline solution [29].

The color changes caused by CEM cement were visible in the first week after applying and it was significantly lower than WMTA throughout the experiment. The results for this group did not vary with those of CH and the control group. 

The lower staining characteristics in comparison with MTA, is probably a result of their different compositions. The results of the energy dispersive X-ray analysis (EDXA) revealed that lime is a predominant component of CEM cement, and other constituents are considerably different from MTA, except for some trace elements. Also Electron probe microanalysis results show that unlike WMTA, bismuth oxide and iron oxide are not present in CEM cement [29]. These oxides as mentioned previously, cause a dark shade in MTA, but CEM cement mass stayed white and opaque after 5 months. The lightness of the tooth considerably decreased after removal the material which is probably due to its opacity. This finding was similar to that of CH group.

The fact that the present study was carried out *in vitro* and lack of previous clinical studies on the subject, indicates the need to evaluate CEM cement color change potential *in vivo* and in contact with vital tissues as well.

## Conclusion

Clinically perceptible crown discoloration was detected from the first week of material application in all the endodontic materials. *ΔE* values presented significantly higher color changes for WMTA in comparison with CEM cement and CH. The discoloration induced by CEM cement was comparable to the control group or CH.
